# Milk as a Diet

**Published:** 1892-07

**Authors:** Geo. E. Newell


### MILK AS A DIET.

Reader, did you ever consider what a complete and superior diet milk was for the human stomach? Used as it is in almost every household in the land, in conjunction with other foods, its real nutritive value is not always appreciated by the laity. Physicians of the highest repute strongly recommend its use for a form of nourishment in disorders where other food would be prohibited. It is so easily digestible, and at the same time so nourishing that very weak stomachs will assimilate it. Milk can by the following formula be digested before it enters the stomach, and thus be rapidly taken up by the blood as nourishment without exciting the action of a perhaps dyspeptic organ: Take of the extract of pancreatine five grains, and of bicarbonate of soda fifteen grains, and add to one pint of fresh milk and a gill of water. Heat the whole to 100° Fahrenheit, and hold at that temperature forty-five minutes; then cool quickly, and it is ready for drinking. This preparation has a slightly bitter taste, and cannot be coagulated by any acid. It is digested milk, and for feeble stomachs it makes an excellent food.

While manufacturers are seeking to extend the consumption of cheese and butter, the consumption of milk is taking care of itself, and it is increasing especially in the cities and towns all over the land. To what cause can this be attributed ? One thing, the good qualities of milk are becoming better known, and then there is far less adulteration of the product than formerly. This is not because the average milkman has reformed especially, but because the laws of inspection are becoming more thorougly enforced. Consumers have become aware of this, aud they know that they can obtain better milk than formerly and at just as low a price.

Our observation of city milk consumption has made us confident of this fact, and it is a pleasing feature of the results of honest dairy work. Do not be afraid to drink all the good sweet milk you want. Physicians recommend it, and dyspeptic stomachs endorse it. What better evidence is needed of its nutritive and valuable qualities as a diet ? It is the healthy naturally flavored milk that is to be recommended. Tainted or stable-flavored milk is not fitted to make even good pork, much less to be used on the table. We do not go so far as to claim that milk is a panacea for all digestive woes, but we do say that it has excellent and enduring qualities as a diet not possessed by other foods, and that it seldom irritates the dyspeptic stomach.—*Geo. E. Newell, in Prairie Farmer.*

